# The Therapeutic Relationship in E-Therapy for Mental Health: A Systematic Review

**DOI:** 10.2196/jmir.2084

**Published:** 2012-08-02

**Authors:** Madalina Sucala, Julie B Schnur, Michael J Constantino, Sarah J Miller, Emily H Brackman, Guy H Montgomery

**Affiliations:** ^1^Department of Oncological SciencesMount Sinai School of MedicineNew York, NYUnited States; ^2^Department of Clinical Psychology and PsychotherapyBabes-Bolyai UniversityCluj-NapocaRomania; ^3^Department of PsychologyUniversity of Massachusetts AmherstAmherst, MAUnited States

**Keywords:** e-Therapy, therapeutic relationship, therapeutic alliance, common factors in psychotherapy

## Abstract

**Background:**

E-therapy is defined as a licensed mental health care professional providing mental health services via e-mail, video conferencing, virtual reality technology, chat technology, or any combination of these. The use of e-therapy has been rapidly expanding in the last two decades, with growing evidence suggesting that the provision of mental health services over the Internet is both clinically efficacious and cost effective. Yet there are still unanswered concerns about e-therapy, including whether it is possible to develop a successful therapeutic relationship over the Internet in the absence of nonverbal cues.

**Objective:**

Our objective in this study was to systematically review the therapeutic relationship in e-therapy.

**Methods:**

We searched PubMed, PsycINFO, and CINAHL through August 2011. Information on study methods and results was abstracted independently by the authors using a standardized form.

**Results:**

From the 840 reviewed studies, only 11 (1.3%) investigated the therapeutic relationship. The majority of the reviewed studies were focused on the therapeutic alliance—a central element of the therapeutic relationship. Although the results do not allow firm conclusions, they indicate that e-therapy seems to be at least equivalent to face-to-face therapy in terms of therapeutic alliance, and that there is a relationship between the therapeutic alliance and e-therapy outcome.

**Conclusions:**

Overall, the current literature on the role of therapeutic relationship in e-therapy is scant, and much more research is needed to understand the therapeutic relationship in online environments.

## Introduction

As of 2011, 78.3% of the US population had Internet access [1]. The widespread use of the Internet has affected mental health care delivery, with a rapid expansion of e-therapy [2]. E-therapy is defined as “a licensed mental health care professional providing mental health services via e-mail, video conferencing, virtual reality technology, chat technology, or any combination of these” [3].

Although there is growing evidence that e-therapy is effective for a variety of conditions [2,4-6], researchers have expressed concerns about e-therapy use [7,8]. One of the primary concerns about e-therapy is related to the perceived difficulty of developing an effective therapeutic relationship in the absence of nonverbal cues [6].

Extensive literature on face-to-face psychotherapy indicates that the therapeutic relationship accounts for more variability in psychotherapy outcomes than do specific therapy ingredients [9-11]. Given the crucial role of the therapeutic relationship in face-to-face interventions, it is important to assess the role of therapeutic relationship in e-therapy as well.

Although e-therapy research began over 15 years ago, there has been no attempt to review the findings pertaining to the status of the therapeutic relationship in online interventions. Heeding the guidelines published by the American Psychological Association (Division 29), which state that descriptions of effective psychotherapies that do not mention the therapeutic relationship are “seriously incomplete and potentially misleading on both clinical and empirical grounds” [12], it is imperative to investigate systematically the status of the therapeutic relationship in e-therapy. This paper represents the first attempt to summarize and review the existing findings. More specifically, the review examined (1) how the therapeutic relationship is being assessed in e-therapy, (2) patients’ satisfaction with the therapeutic relationship in e-therapy, (3) differences in the therapeutic relationship between e-therapy and face-to-face therapy, (4) factors that may influence the therapeutic relationship in e-therapy, and (5) the relationship between the therapeutic relationship and treatment outcome in e-therapy.

## Methods

### Search Strategy

We searched 3 electronic databases (PubMed, PsycINFO, and CINAHL) from their respective inceptions through August 2, 2011. For PubMed, the search terms were (counseling[MeSH] OR psychotherapy[MeSH]) AND Internet[MAJR]). The search was limited by language (the paper had to be in English), by methodology (the study had to be a clinical trial; randomized controlled trial; clinical trial, phase 1; clinical trial, phase 2; clinical trial, phase 3; clinical trial, phase 4; comparative study; controlled clinical trial; or a technical report), and by sample (human subjects). This search, with these limits, and taking only the items with an abstract, yielded a total of 468 abstracts.

For PsycINFO, the major search terms were ([exp counseling OR exp psychotherapy] AND exp Internet). The search was limited by language (the paper had to be in English), by methodology (the study had to be an empirical study, experimental replication, follow-up study, longitudinal study, prospective study, retrospective study, quantitative study, or treatment outcome/randomized clinical trial), by publication type (the study had to be a journal article published in a peer-reviewed journal), and by sample (the study had to be conducted on humans). This search, with these limits, and taking only the items with an abstract, yielded a total of 188 abstracts.

For CINAHL, the major search terms were ([MH psychotherapy OR MH counseling] AND MH Internet). The search was limited by language (the paper had to be in English) and by publication type (the study had to be a peer-reviewed research article). This search, with these limits, and taking only the items with an abstract, yielded a total of 184 abstracts.

### Selection Strategy

We carefully screened the abstracts of all articles identified by the electronic searches (840 in total) to determine whether the abstracts met the following inclusion criteria: (1) described an intervention study that empirically assessed the effects of e-therapy on an outcome (excluding qualitative studies, survey studies, reviews, meta-analyses, etc), and (2) reported data relevant to the therapeutic relationship. Specifically, abstract text had to use the word *relationship *or *alliance *to be included in the review. Interventions had to be consistent with the above definition of e-therapy. This excluded interventions that were described as self-help, peer-led groups, online communities, or volunteer-led online support. If a given study had multiple intervention groups, at least one intervention group had to meet the e-therapy definition. There were no inclusion or exclusion criteria regarding the focus of the treatment.

**Figure 1:**
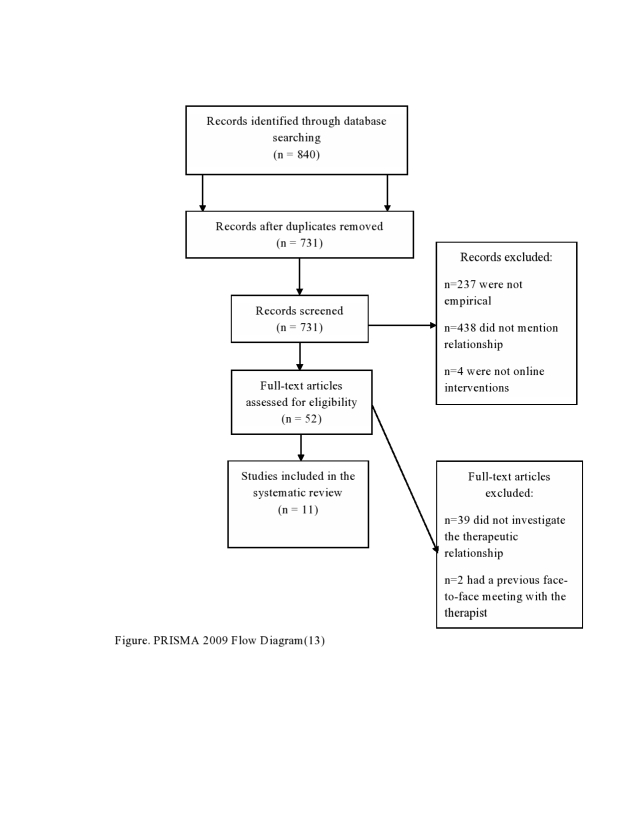
PRISMA 2009 Flow Diagram.

Based on these criteria, the number of eligible abstracts was reduced from 840 abstracts to 56 abstracts. Figure 1 details reasons for exclusion [13]. The 56 manuscripts were obtained and read in full, independently, by two of the authors (MS and SJM). They completed a standardized form assessing the above-listed criteria. Any lack of consensus was discussed with JBS and GHM until consensus was reached.

### Data Abstraction and Study Characteristics

We included 11 of the 56 studies in the review (Table 1). Each of the 11 papers was abstracted independently by MS and SJM. The data abstraction form included (1) authors and publication date, (2) the study sample (eg, demographic characteristics), (3) the intervention and the interventionists, (4) study design elements, (5) the therapeutic relationship element studied and how it was measured (eg, scale), and (6) the findings pertaining to the therapeutic relationship. Any discrepancies were discussed among the authors (MS and SJM) with reference to the original manuscript until consensus was reached.

The quality of each study was evaluated independently by MS and SJM according to the following eight validity criteria, which were adapted from the Consolidated Standards of Reporting Trials (CONSORT) guidelines [14,15] and Delphi criteria list [16]: randomization; allocation concealment; blinding of outcome assessments; comparability of groups at baseline; withdrawals; handling of dropouts in analyses; use of intention-to-treat analysis; and multiple follow-up assessments. Scores were given, with 1 point allocated for each criterion satisfied (range 0-8 points). The interrater reliability between them was .84, indicating strong agreement [17]. Any discrepancies were discussed (with JBS and GHM) with reference to the original manuscript until consensus was reached.

Although some quantitative data were available, there were insufficient data for formal comprehensive meta-analyses. Therefore, we report effect sizes where possible and informative.

## Results

Among the included studies, investigating the therapeutic relationship was a primary objective for 6 [4,6,18-21], whereas for the other 5 studies, the assessment of the therapeutic relationship was a secondary outcome.

### Study and Participant Characteristics

Table 1 summarizes design characteristics and quality scores. The quality scores for the studies ranged from 0 to 7 out of a maximum of 8 points. Because blinding of participants to the type of intervention is often practically impossible in psychosocial interventions, as participants must actively engage in them, no study could receive a perfect score of 8. A total of 6 studies were described as randomized; 2 studies used only pre-post comparisons to analyze data pertaining to the therapeutic relationship. The other studies had a nonequivalent group design: 2 studies compared e-therapy data with data from previously published studies and 1 study used naturalistic independent samples of participants provided by a youth counseling service. The main limitations for the studies were not comparing groups at baseline [4,20,22,23]; not reporting the use of intention-to-treat analyses or handling of missing data [4,6,18,21]; and not using follow-up assessments [4,6,18,20,22,24].

The main therapeutic approach used in the analyzed studies was cognitive behavioral therapy (CBT) (*k *= 9). E-therapists were psychologists and psychotherapists (*k *= 6), psychology students (*k *= 4), and counselors (*k *= 1). Overall, the dose of e-therapy ranged from 1 session to 11 weeks, with a mean of 7.75 (SD 2.37) weeks. Communication between therapist and patient was conducted via asynchronous email and website postings (*k *= 8), synchronous website text exchange (*k *= 1), synchronous chat (*k *= 1), or a combination of asynchronous email and synchronous chat (*k *= 1).

**Table 1:** Study design characteristics^a^.

Authors	Sample size	E-therapy communication modality	Therapists	Treatment length	Study design	Study quality^b^
Cook and Doyle [6]	15	Email and chat, both asynchronous and synchronous communication^c^	1 PhD, 3 masters level, and 1 masters student	1–5 sessions	Nonequivalent groups design; compared e-therapy versus previous normative data from face-to-face counseling	1
King et al [18]	186	Website postings, synchronous communication	“Trained counselors”	1 session, with a typical session duration of 50–80 minutes	Nonequivalent group design; e-therapy versus telephone counseling	2
Kiropoulos et al [24]	86	Website and email, asynchronous communication	9 registered psychologists, 1 probationary psychologist	8 weeks, weekly assignments	RCT^d ^design; e-CBT^e ^versus face-to-face CBT	6
Klein et al [22]	16	Website and email, asynchronous communication	6 registered psychologists, 1 probationary registered psychologist	10 weeks, weekly assignments	RCT design; pre- to posttreatment comparisons	3
Klein et al [23]	22	Website and email, asynchronous communication	6 registered psychologists, 1 probationary registered psychologist	10 weeks, weekly assignments	RCT design; pre- to posttreatment comparisons	4
Knaevelsrud and Maercker [20]	48	Email, asynchronous communication	2 psychologists	5 weeks, 2 weekly 45-minute writing assignments	RCT; e-CBT versus waiting list	3
Knaevelsrud and Maercker [19]	96	Email, asynchronous e-communication	2 clinical psychologists at the doctoral level	5 weeks, 2 weekly 45-minute writing assignments	RCT; e-CBT versus waiting list	6
Reynolds et al [4]	17	Email, asynchronous communication	16 psychotherapists (62.5% qualified to work in the United States)	Not reported	Nonequivalent group design; e-therapy versus data from prior face-to-face studies	0
Ruwaard et al [25]	239	Email, asynchronous communication	25 doctoral and 1 postgraduate student in clinical psychology	7 weeks, 5 hours of therapist time	RCT; e-CBT versus waiting list	6
Ruwaard et al [26]	54	Website, asynchronous communication	18 graduate-level clinical psychologists and 6 therapists	11 weeks, 22–44 hours of patient time and 7–14 hours of therapist time	RCT; e-CBT versus waiting list	6
Trautmann and Kroner-Herwig [21]	18	Chat, synchronous communication	3 clinical psychology graduate students	6 weeks, a weekly chat with the therapist	RCT; e-CBT versus e-psychoeducation	4

^a ^The table presents the information about the studies’ characteristics; not all of the studies provided a detailed description of the methods.

^b ^Score for number of validity criteria satisfied (range 1–8).

^c ^Synchronous communication between therapist and patient takes places in real time, in a same-time/different-place mode (eg, chat); asynchronous communication takes place over a period of time through a different-time/different-place mode (eg, email).

^d ^Randomized controlled trial.

^e ^Cognitive behavioral therapy.

Table 2 summarizes participants’ characteristics by study. The participants were receiving e-therapy for a variety of problems, including mental health diagnosis (eg, posttraumatic stress disorder, *k *= 4; depression, *k *= 1; and panic disorder and agoraphobia, *k *= 1), psychological distress related to medical problems (eg, headaches, *k *= 1), work-related distress (*k *= 1), general distress (*k *= 1), and other self-reported presenting problems (eg, symptoms of depression, symptoms of anxiety, stress, relationship issues, or childhood abuse; *k *= 2). Participants were both adolescents (*k *= 2) and adults (*k *= 9). A majority of the adult patients were women (at least 60% across the studies) with a high level of education (at least 44% across the studies completed college).

**Table 2:** Participants’ characteristics^a^.

Authors	Presenting problem	Age (years), mean (SD)	Gender	Education	Race/ ethnicity
Cook and Doyle [6]	Relationship issues and depression	41.40 (15.99)	93%women	All participants completed at least high school	“Primarily white”
King et al [18]	Distress	14.25 (no SD provided)	80.1% women	Not reported	Not reported
Kiropoulos et al [24]	Panic disorder and agoraphobia	38.96 (11.13)	72.1% women	Mean education level 12.53 (SD 6.14) years	Not reported
Klein et al [22]	Posttraumatic stress disorder	48 (no SD provided)	81.2% women	Mean education level 13 years (no SD provided)	Not reported
Klein et al [23]	Posttraumatic stress disorder	45.8 (no SD provided)	77.2% women	Mean education level 13.3 (SD 3.5) years	Not reported
Knaevelsrud and Maercker [20]	Posttraumatic stress disorder	35 (no SD provided)	92% women	55% had a university degree	Not reported
Knaevelsrud and Maercker [19]	Posttraumatic stress disorder	35 (no SD provided)	90% women	44% had a university degree	Not reported
Reynolds et al [4]	Depression, stress, anxiety, and childhood abuse	Median 39	71% women	94.1% completed high school	82% Caucasian
Ruwaard et al [25]	Work-related stress	44 (8)	60% women	84% had a university degree	Not reported
Ruwaard et al [26]	Depression	21 (10)	69% women	65% had a university degree	Not reported
Trautmann and Kroner-Herwig [21]	Headache	13.4 (2.6)	Not reported	Not reported	Not reported

^a ^The table presents the information about the patients’ characteristics that the studies provided; not all the studies provided the full range of demographic information.

### Assessment of the Therapeutic Relationship in E-Therapy

Table 3 presents, by study, the characteristics of the measures used to assess the therapeutic relationship.

A total of 3 of the studies [22-24] used the Therapist/Therapeutic Alliance Questionnaire, a modified version of the Helping Alliance Questionnaire [29]. The scale requires the participants to estimate the degree to which the therapeutic alliance with their therapist was helpful.

Cook and Doyle [6] used the Working Alliance Inventory [27]. The scale is based on Bordin’s concept of therapeutic alliance: therapist-patient agreement on therapeutic goals; therapist-patient agreement on therapeutic tasks, and the quality of the emotional bond between the therapist and the patient [35,36]. Two other studies [19,20] used the short version of the Working Alliance Inventory [30].

King et al [18] used the Therapeutic Alliance Scale [28]. The scale evaluates the overall therapeutic alliance with 3 subcomponents: mutual liking between therapist and patient, collaboration between therapist and patient, and resistance (ie, resistance to the treatment program).

Reynolds et al [4] used the Agnew Relationship Measure-Short Form [31]. The scale evaluates the overall therapeutic alliance with 3 subcomponents: bond and partnership, confidence (defined as the confident collaboration between patient and therapist), and openness (defined as “the felt freedom to disclose and reveal personal material without fear of censure or embarrassment”) [32].

Trautmann and Kroner-Herwig [21] used an Internet-based patient-therapist alliance/assistance questionnaire [33], which was adapted for use with children and adolescents and to the conditions of e-therapy (eg, “My therapist’s explanations helped and supported me;” “This week I learned something that can help me cope with my headache”). The questionnaire was developed for the purpose of this study, and no other details were provided about this measure.

The other 2 studies included in this review [25,26] investigated patients’ satisfaction with the therapeutic relationship. Both used a scale with 4 items, which assessed the extent to which the participants regarded their relationship with the therapist as being pleasant and personal, whether they perceived the relationship as growing during the treatment, and whether they missed face-to-face contact. No other details were provided about this measure.

**Table 3:** Therapeutic relationship measures and findings.

Authors	Therapeutic relationship measure	Moment of assessment^a^	Measured constructs	Item description and properties	Instrument description and psychometric properties	Patient ratings
Cook and Doyle [6]	Working Alliance Inventory [27]	Third session	Overall therapeutic alliance and 3 subcomponents: (1) agreement on goals, (2) agreement on tasks, (3) bond	36 items scored on a 7-point Likert scale ranging from 1 (*never*) to 7 (*always*). Subscales scores can range from 12 to 84, and total scores can range from 36 to 252. Higher scores reflect more positive ratings of therapeutic alliance.	Good construct validity and high internal consistency on the composite score (.93) as well as for the subscales (.85-.88) [27]	Overall therapeutic alliance, mean 215.07; agreement on task, mean 70.33; agreement on goals, mean 72.27; bond, mean 72.47
King et al [18]	Therapeutic Alliance Scale [28]	Posttreatment	Overall therapeutic alliance and 3 subcomponents: (1) mutual liking, (2) resistance, (3) collaboration	30 items, scored on a 3-point Likert scale (*disagree, somewhat agree, agree*), with scores ranging from 30 to 90, where higher scores indicate more positive perceptions of alliance.	Good internal consistency for the subscales (.83-.90) [28] and for entire measure (.92) [18]	Overall alliance, mean 74.0 (SD 10.4); resistance, mean 24.6 (SD 4.6); mutual liking, mean 26.2 (SD 3.6); collaboration, mean 23.1 (SD 4.8)
Kiropoulos et al [24]	Therapist Alliance Questionnaire [29]	Posttreatment	Overall therapeutic alliance (ie, the degree to which patients experience their therapeutic relationship as being helpful)	17 items rated on a Likert scale ranging from 1 to 7 and summed to produce a total score, which can range from 17 to 102. Higher scores reflect more positive ratings of the therapeutic alliance.	No data were found about the psychometric properties	Mean 83.13 (SD 11.20)
Klein et al [22]	Therapeutic Alliance Questionnaire [29]	Posttreatment	Overall therapeutic alliance (ie, the degree to which patients experience their therapeutic relationship as being helpful)	17 items rated on a Likert scale ranging from 1 to 7 and summed to produce a total score, which can range from 17 to 102. Higher scores reflect more positive ratings of the therapeutic alliance.	No data were found about the psychometric properties	Mean 86.25 (SD 16.23)
Klein et al [23]	Therapeutic Alliance Questionnaire [29]	Posttreatment	Overall therapeutic alliance (ie, the degree to which patients experience their therapeutic relationship as being helpful)	17 items rated on a Likert scale ranging from 1 to 7 and summed to produce a total score, which can range from 17 to 102. Higher scores reflect more positive ratings of the therapeutic alliance.	No data were found about the psychometric properties	Mean 89.18 (SD 15.13)
Knaevelsrud and Maercker [20]	Working Alliance Inventory-short version [30]		Overall therapeutic alliance and 3 subcomponents: (1) agreement on goals, (2) agreement on tasks, (3) bond	12 items scored on a 7-point Likert scale with scores ranging from 1 to 7. Higher scores reflect more positive ratings of therapeutic alliance.	Good internal consistency for the subscales (.90-.92) and for the composite score (.98) [30]	Overall alliance, mean 5.8 (SD 0.62); agreement on goals, mean 5.8 (SD 0.77); agreement on tasks, mean 5.7 (SD 0.80); bond, mean 6.2 (SD 0.75)
Knaevelsrud and Maercker [19]	Working Alliance Inventory-short version [30]	Fourth session	Overall therapeutic alliance and 3 subcomponents: (1) agreement on goals, (2) agreement on tasks, (3) bond	12 items scored on a 7-point Likert scale with scores ranging from 1 to 7. Higher scores reflect more positive ratings of therapeutic alliance.	Good internal consistency for the subscales (.90-.92) and for the composite score (.98) [30]	Overall alliance, mean 5.8 (SD 0.72); agreement on goals, mean 5.8 (SD 0.77); agreement on tasks, mean 5.7 (SD 0.83); bond, mean 6.2 (SD 0.69)
Reynolds et al [4]	Agnew Relationship Measure-Short Form [31]	Fourth session	Overall therapeutic alliance and 3 subcomponents: (1) bond and partnership, (2) confidence, (4) openness	12 items, each rated on a 7-point Likert scale, with higher scores indicating more positive perceptions of alliance.	Good construct validity and high internal consistency, ranging from .83 to .89 [32]	Bond and partnership, mean 5.97 (SD 1.26); confidence, mean 6.19 (SD 1.24); openness, mean 5.27 (SD 1.42)
Ruwaard et al [25]	Treatment satisfaction items [25]	Posttreatment	Aspects of patients’ perceived relationship with their therapists	4 items	The scale was developed for the purpose of the study; no data provided about the psychometric properties	Participants rated the relationship as pleasant (88%) and personal (75%); they perceived the relationship to grow during the treatment (57%); 68% said that they did not miss face-to-face contact
Ruwaard et al [26]	Treatment satisfaction items [25]	Posttreatment	Aspects of patients’ perceived relationship with their therapists	4 items	The scale was developed for the purpose of the study; no data provided about the psychometric properties	Participants rated the relationship as pleasant (88%) and personal (75%); they perceived the relationship to grow during the treatment (57%); 89% said that they did not miss face-to-face contact
Trautmann and Kroner-Herwig [21]	Internet-based questionnaire on patient-trainer alliance/assistance scale [33]	Posttreatment	Patient–trainer alliance	The scale ranges from 0 to 3, with higher scores indicating more positive perceptions of alliance.	The scale was developed for the purpose of the study; no data provided about the psychometric properties	E-CBT^b^, median 2.8, range 2–3; e-psychoeducation, median 2.7, range 2–3

^a ^If multiple assessment points were used, we present the data for the earliest point of assessment, since previous studies showed that the level of alliance, regardless of the length of therapy, is established within the first sessions, recommending that alliance be assessed at the beginning of therapy [34].

^b ^Cognitive behavioral therapy.

### Patients’ Satisfaction With the Therapeutic Relationship in E-Therapy

Ruwaard and colleagues [25] investigated the impact of e-therapy on work-related stress on a sample of 239 participants. Results indicated that participants rated the e-therapy relationship as being pleasant (210/239, 88%) and personal (179/239, 75%); 136 of 239 (57%) perceived the relationship to grow during the treatment, and 163 of 239 (68%) said that they did not miss face-to-face contact.

A second study conducted by Ruwaard and colleagues [26] investigated the impact of e-therapy on depression on a sample of 54 participants. The study yielded similar results to those previously described, in that the majority of the participants rated the e-therapy relationship as being pleasant (47/54, 87%) and personal (42/54, 78%), perceived the relationship to be growing during the treatment (42/54, 78%), and reported that they did not miss face-to-face contact (48/54, 89%). The results of the studies indicated that patients offered high ratings for therapeutic relationship in e-therapy (see Table 3).

### Differences in the Therapeutic Relationship Between E-Therapy and Face-to-Face Therapy

A total of 3 studies investigated differences in the therapeutic alliance between e-therapy and face-to-face therapy [4, 6, 24]. Results were mixed, with 2 studies showing no significant differences in therapeutic alliance (eg, overall alliance and various subscales) between e-therapy and face-to-face therapy, and 1 study showing higher scores for therapeutic alliance in e-therapy than in face-to-face therapy.

Kiropoulos and colleagues [24] investigated whether the therapeutic alliance in e-therapy is different from face-to-face therapy in a study comparing e-CBT with face-to-face CBT for panic disorder and agoraphobia. Results indicated that there was no significant difference between groups for therapeutic alliance score (*t*_47 _= –1.02, *P *= .31, *d *= 0.29; according to Cohen [37], an effect size of 0.2 to 0.3 represents a small effect, around 0.5 represents a medium effect, and 0.8 or greater represents a large effect).

Reynolds et al [4] compared e-therapy data with data from previously published face-to-face studies. Results indicated that patients gave high ratings for therapeutic alliance in e-therapy, with the means for the subscales of bond and partnership between therapist and patient (mean 5.97, SD 1.26) and confident collaboration between therapist and patient (mean 6.19, SD 1.24) within the range of reported means for previous face-to-face therapy studies. The mean for openness (mean 5.27, SD 1.42) in e-therapy was below the range of means from the prior face-to-face studies. However, it is important to note that no test for statistical significance was performed.

Cook and Doyle [6] investigated whether the therapeutic alliance in e-therapy is different from face-to-face therapy in a sample of 15 participants. Results indicated that the overall working alliance scores (*t*_14 _= 3.03, *P *< .001, *d *= 0.60) and the agreement between therapist and patient on the therapy goals subscale scores (*t*_14 _= 2.30, *P *= .03, *d *= 0.79) were significantly higher in e-therapy than in face-to-face interventions, with medium to large effect sizes. The agreement between therapist and patient on tasks (*t*_14 _= 1.26, *P *= .22, *d *= 0.22) and the bond between therapist and patient were rated higher as well (*t*_14 _= 1.62, *P *= .12, *d *= 0.33), although the difference did not reach statistical significance and the effect sizes were small.

### Factors That May Influence the Therapeutic Relationship in E-Therapy

A total of 2 studies investigated factors that might influence the therapeutic relationship in e-therapy [6, 20]. Knaevelsrud and Maercker [20] reported an inverse relationship between pretreatment symptom severity and therapeutic alliance ratings, such that patients who experienced more severe anxiety symptoms at the beginning of treatment tended to give lower ratings for the bond between therapist and patient subscale (*r *= –.34, *P *< .05, *d *= 0.72). There was an overall tendency for an inverse relationship between pretreatment anxiety and depression symptoms, and agreement on goals and task subscales ratings, but the correlations did not reach statistical significance and the effect sizes were small to moderate (all *P *> .05, all *d *< 0.40).

Cook and Doyle [6] investigated the impact of communication modality on the therapeutic relationship. Their results did not reach statistical significance. However, they reported that participants who used chat as the primary mode of communication (eg, as opposed to email) had consistently higher means for the therapeutic alliance than did participants who used email (overall alliance, *t*_13 _= 1.54, *P *= .10, *d *= 1.13; agreement on task, *t*_13 _= 0.89, *P *= .37, *d *= 0.54; agreement on goals, *t*_13 _= 1.54, *P *= .12, *d *= 1.09; bond, *t*_13 _= 1.92, *P *= .07, *d *= 1.19), obtaining medium to large effect sizes. Participants who used more than one modality of communication (eg, email plus chat) had higher ratings for the therapeutic alliance than did participants who used only one modality of communication (overall alliance, *t*_13 _= 1.87, *P *= .08, *d *= 1.02; agreement on tasks, *t*_13 _= 1.67, *P *= .11, *d *= 0.91; agreement on goals, *t*_13 _= 1.40, *P *= .18, *d *= 0.76; bond, *t*_13 _= 1.67, *P *= .11, *d *= 0.91). However, it should be noted that these results were based on comparisons made on very small samples of participants (eg, participants who used chat as a primary communication mode, n = 3, versus participants who used email as a primary communication mode, n = 12).

### Is the Quality of the Therapeutic Relationship Linked to Treatment Outcome in E-Therapy?

A total of 3 studies investigated the impact of the therapeutic alliance on treatment outcome [18-20]. Knaevelsrud and Maercker [20] investigated the relationship between working alliance and the outcome of e-therapy for patients with posttraumatic stress disorder. Results showed that the composite score for therapeutic alliance correlated positively with residual gain scores for anxiety (*r *= .33, *P *< .05, *d *= 0.69), which indicates that patients who rated the alliance as better had greater reduction of their anxiety scores at posttreatment.

Knaevelsrud and Maercker [19], in a later study investigating the impact of e-therapy on posttraumatic stress disorder, found that overall patient-rated working alliance at posttreatment predicted 15% of the variance in the scores for posttraumatic stress symptoms (adjusted *R* ^2 ^= .148, *F*_2,39 _= 8.15, *P *< .001), obtaining a large effect size.

King and colleagues [18] investigated the impact of online versus telephone counseling for adolescents. Their results revealed a modest trend toward a relationship between the collaboration subscale scores and posttreatment distress (beta = 0.25, *t *= 1.83, *P *= .07, *d *= 0.14) and a significant effect of the resistance subscale on posttreatment distress (beta = 1.21, *t *= 2.40, *P *< .05, *d *= 0.19).

## Discussion

To our knowledge, this study is the first to summarize and review the findings on the role of the therapeutic relationship in e-therapy. The most striking finding was the limited number of studies investigating the therapeutic relationship. Of the 840 reviewed studies, only 11 (1.3%) addressed and investigated the issue of the therapeutic relationship, and of these, only 6 investigated the therapeutic relationship as a primary objective. In other words, the results indicate that, although the therapeutic relationship is considered to be an important common factor operating across all psychotherapies [34,38], the study of the therapeutic relationship appears to have been largely ignored in the e-therapy literature.

The reviewed studies have the merit of providing a first glimpse into the role of the therapeutic relationship in e-therapy. However, due to the small number of studies and to their methodological limits, the findings must be interpreted with caution.

### Study and Participant Characteristics

The methodological limits of the studies included the lack of suitable control groups (nonrandom allocation or nonequivalent group design), lack of pretest information, poor reporting and handling of dropouts in the analyses, and more generally an often incomplete presentation of results (eg, not reporting standard deviations, not reporting effect sizes). As research moves forward, it is important for future studies to adhere to the standards of conducting and reporting psychosocial interventions [13]. Improved reporting will lead to the enrichment of systematic reviews and allow for better-informed treatment decision making among practitioners. Another issue that might limit enthusiasm for the findings is that the majority of studies were affected by a selection bias. The recruitment was performed through webpages or email announcements, which already rely on a certain familiarity with the use of the Internet. This is a particularly important methodological limitation, since previous studies indicated that, the more familiar participants are with Internet-based contact, the more positively they judge Internet-based contact to be [39]. Future studies should clarify the role of Internet familiarity in the therapeutic relationship in e-therapy.

As for the studies’ characteristics, it is interesting to note that the main therapeutic approach was CBT, which easily lends itself to standardized instructions and short-term, manualized approaches. Because almost all studies included in this review used a CBT approach, it is difficult to infer the status of therapeutic relationship in online interventions that use therapeutic approaches that are less structured.

The overwhelming majority of participants were women, consistent with previous research that has found that more women than men use the Internet for mental health information and services [40]. Participants tended to be highly educated. Not all the studies provided information about other important demographics, such as race and ethnicity, but it is interesting to note that in the 2 studies that reported this information, the participants were primarily white. These results are consistent with previous findings, indicating that health information seeking over the Internet is more prevalent among white, educated women, and that mental health information seeking in particular tends to have the same type of consumers [40]. To determine the appropriateness of e-therapy and to investigate the status of the therapeutic relationship in online environments across ethnic groups, future research should include more diverse samples of patients.

### Assessment of the Therapeutic Relationship in E-Therapy

The majority of the studies focused on a specific element of the therapeutic relationship, namely the therapeutic alliance. The studies used a variety of measures to assess the therapeutic alliance, defining the concept by the instrument used to measure it. In that sense, as Norcross [34] suggested, “instrumentation defines the construct.” In addition, some of the studies used measures that had been created on an ad hoc basis. If progress is to be made in this field, future studies should reach toward a consensus by using validated measures based on supported conceptualizations of what therapeutic alliance is [34,41].

As for the timing of the assessment, the majority of the studies investigated the therapeutic alliance at the end of therapy. Previous studies indicate that the level of alliance, regardless of the length of therapy, is established within the first sessions [34]. Meta-analytic studies also revealed that early alliance is more predictive of outcome than is alliance assessed later in therapy [42]. Accordingly, it is recommended that future e-therapy studies assess alliance at the beginning of the treatment.

### Differences in the Therapeutic Relationship Between E-Therapy and Face-to-Face Therapy

A surprising finding, given the previous concerns related to the lack of nonverbal cues in e-therapy, is that e-therapy seems to be at least equivalent to face-to-face therapy in terms of the therapeutic relationship (more specifically therapeutic alliance). Although very promising and clearly worthy of attention, this line of research is in its infancy, and further research is needed to draw firm conclusions.

### Factors That May Influence the Therapeutic Relationship in E-Therapy

Although the results do not allow firm conclusions to be drawn, it seems that investigating factors such as communication modality and pretreatment symptom severity as moderators of the therapeutic relationship might be a fruitful direction of research. In addition, all of the studies included in this review used text-based communication methods; thus, it would be important to investigate the status of the therapeutic relationship when the communication modality includes video conferencing (eg, through Skype), where the verbal cues are not missing and the communication is synchronous.

### Is the Quality of the Therapeutic Relationship Linked to Treatment Outcome in E-Therapy?

The 3 studies investigating the impact of the therapeutic alliance on treatment outcome indicate that these two factors have a positive relationship. This avenue of research should be further pursued, as it offers a hint that the beneficial effects of this therapeutic relationship element are not restricted to face-to-face therapies.

### Limitations, Conclusions, and Future Directions

The present review has limitations. First, it was based on searches in three databases—PubMed, PsycINFO, and CINAHL—and was limited to published papers in English. It is possible that additional relevant papers exist outside of the present sample of papers. Second, the reviewed abstracts were required to report the assessment of the therapeutic relationship. It is possible that papers exist for studies in which investigating the relationship was not a main goal, and thus their abstracts might not refer to it. Future work may include more languages, include unpublished manuscripts, and use a wider variety of search terms to confirm the generalizability of the present conclusions. Additionally, once the literature grows large enough, a formal meta-analysis should be conducted to estimate the overall effect size for both the impact of the relationship on psychotherapy outcome and differences in the relationship between face-to-face therapy and e-therapy. Future meta-analyses would also have the potential to explore moderators of relationship effects and would be an important step forward for the field.

Overall, this review summarizes research to date on the therapeutic relationship in e-therapy. If relationship is considered a common factor in successful psychotherapy, it should become commonly studied in e-therapy as well. Looking to the future, we hope that the present findings will spur investigation into the role of the therapeutic relationship in e-therapy.

## Abbreviations

CBT: cognitive behavioral therapy

## References

[1] (2012). Miniwatts Marketing Group.

[2] Barak A, Hen L, Boniel-Nissim M, Shapira N (2008). A comprehensive review and a meta-analysis of the effectiveness of Internet-based psychotherapeutic interventions. J Technol Hum Serv.

[3] Manhal-Baugus M (2001). E-therapy: practical, ethical, and legal issues. Cyberpsychol Behav.

[4] Reynolds DJ, Stiles WB, Grohol JM (2006). An investigation of session impact and alliance in internet based psychotherapy: preliminary results. Couns Psychother Res.

[5] Mallen MJ, Vogel DL, Rochlen AB, Day SX (2005). Online counseling: reviewing the literature from a counseling psychology framework. Couns Psychol.

[6] Cook JE, Doyle C (2002). Working alliance in online therapy as compared to face-to-face therapy: preliminary results. Cyberpsychol Behav.

[7] Rochlen AB, Zack JS, Speyer C (2004). Online therapy: review of relevant definitions, debates, and current empirical support. J Clin Psychol.

[8] Wells M, Mitchell KJ, Finkelhor D, Becker-Blease KA (2007). Online mental health treatment: concerns and considerations. Cyberpsychol Behav.

[9] Lambert MJ, Barley DE (2001). Research summary on the therapeutic relationship and psychotherapy outcome. Psychotherapy.

[10] Norcross JC (2002). Empirically supported therapy relationships. Norcross JC, editor. Psychotherapy Relationships That Work: Therapist Contributions and Responsiveness to Patients.

[11] Wampold BE (2001). The Great Psychotherapy Debate: Models, Methods, and Findings.

[12] Ackerman SJ, Benjamin LS, Beutler LE, Gelso CJ, Goldfried MR, Hill C, Lambert MJ, Norcross JC, Orlinsky DE, Rainer J (2001). Empirically supported therapy relationships: conclusions and recommendations of the Division 29 Task Force. Psychotherapy.

[13] Moher D, Liberati A, Tetzlaff J, Altman DG, PRISMA Group (2009). Preferred reporting items for systematic reviews and meta-analyses: the PRISMA statement. Ann Intern Med.

[14] Altman DG, Schulz KF, Moher D, Egger M, Davidoff F, Elbourne D, Gøtzsche PC, Lang T, CONSORT GROUP (Consolidated Standards of Reporting Trials) (2001). The revised CONSORT statement for reporting randomized trials: explanation and elaboration. Ann Intern Med.

[15] Moher D, Schulz KF, Altman DG (2001). The CONSORT statement: revised recommendations for improving the quality of reports of parallel-group randomised trials. Lancet.

[16] Verhagen AP, de Vet HC, de Bie RA, Kessels AG, Boers M, Bouter LM, Knipschild PG (1998). The Delphi list: a criteria list for quality assessment of randomized clinical trials for conducting systematic reviews developed by Delphi consensus. J Clin Epidemiol.

[17] Landis JR, Koch GG (1977). The measurement of observer agreement for categorical data. Biometrics.

[18] King R, Bambling M, Reid W, Thomas I (2006). Telephone and online counselling for young people: a naturalistic comparison of session outcome, session impact and therapeutic alliance. Couns Psychother Res.

[19] Knaevelsrud C, Maercker A (2007). Internet-based treatment for PTSD reduces distress and facilitates the development of a strong therapeutic alliance: a randomized controlled clinical trial. BMC Psychiatry.

[20] Knaevelsrud C, Maercker A (2006). Does the quality of the working alliance predict treatment outcome in online psychotherapy for traumatized patients?. J Med Internet Res.

[21] Trautmann E, Kroner-Herwig B (2008). Internet-based self-help training for children and adolescents with recurrent headache: a pilot study. Behav Cogn Psychother.

[22] Klein B, Mitchell J, Gilson K, Shandley K, Austin D, Kiropoulos L, Abbott J, Cannard G (2009). A therapist-assisted Internet-based CBT intervention for posttraumatic stress disorder: preliminary results. Cogn Behav Ther.

[23] Klein B, Mitchell J, Abbott J, Shandley K, Austin D, Gilson K, Kiropoulos L, Cannard G, Redman T (2010). A therapist-assisted cognitive behavior therapy internet intervention for posttraumatic stress disorder: pre-, post- and 3-month follow-up results from an open trial. J Anxiety Disord.

[24] Kiropoulos LA, Klein B, Austin DW, Gilson K, Pier C, Mitchell J, Ciechomski L (2008). Is internet-based CBT for panic disorder and agoraphobia as effective as face-to-face CBT?. J Anxiety Disord.

[25] Ruwaard J, Lange A, Bouwman M, Broeksteeg J, Schrieken B (2007). E-mailed standardized cognitive behavioural treatment of work-related stress: a randomized controlled trial. Cogn Behav Ther.

[26] Ruwaard J, Schrieken B, Schrijver M, Broeksteeg J, Dekker J, Vermeulen H, Lange A (2009). Standardized web-based cognitive behavioural therapy of mild to moderate depression: a randomized controlled trial with a long-term follow-up. Cogn Behav Ther.

[27] Horvath AO, Greenberg LS (1986). Development of the Working Alliance Inventory. Greenberg LS, Pinsoff WM, editors. The Psychotherapeutic Process: A Research Handbook.

[28] Bickman L, Vides de Andrade AR, Lambert EW, Doucette A, Sapyta J, Boyd AS, Rumberger DT, Moore-Kurnot J, McDonough LC, Rauktis MB (2004). Youth therapeutic alliance in intensive treatment settings. J Behav Health Serv Res.

[29] Luborsky L, McLellan AT, Woody GE, O'Brien CP, Auerbach A (1985). Therapist success and its determinants. Arch Gen Psychiatry.

[30] Tracey TJ, Kokotovic AM (1989). Factor structure of the working alliance inventory. Psychol Assess.

[31] Stiles WB, Hardy GE, Cahill J (2003). The short ARM (a short form of the Agnew Relationship Measure).

[32] Cahill J, Stiles WB, Barkham M, Hardy GE, Stone G, Agnew-Davies R, Unsworth G (2012). Two short forms of the Agnew Relationship Measure: the ARM-5 and ARM-12. Psychother Res.

[33] Krampen G, Wald B (2001). Kurzinstrumente fur die Prozessevaluation und adaptive Indikation in der Allgemeinen und Differentiellen Psychotherapie und Beratung. Diagnostica.

[34] Norcross JC, Norcross JC (2011). Psychotherapy Relationships That Work: Evidence-Based Responsiveness. 2nd edition.

[35] Bordin ES (1976). The generalizability of the psychoanalytic concept of the working alliance. Psychotherapy.

[36] Bordin ES (1994). Theoryresearch on the therapeutic working alliance: new directions. Horvath AO, Greenberg LS, editors. The Working Alliance: Therory, Research, and Practice.

[37] Cohen J (1988). Statistical Power Analysis for the Behavioral Sciences. 2nd edition.

[38] Castonguay LG, Constantino MJ, Holtforth MG (2006). The working alliance: Where are we and where should we go?. Psychotherapy (Chic).

[39] Mallen MJ, Day SX, Green MA (2003). Online versus face-to-face conversations: an examination of relational and discourse variables. Psychotherapy.

[40] Powell J, Clarke A (2006). Internet information-seeking in mental health: population survey. Br J Psychiatry.

[41] Hatcher RL, Barends A, Hansell J, Gutfreund MJ (1995). Patients' and therapists' shared and unique views of the therapeutic alliance: an investigation using confirmatory factor analysis in a nested design. J Consult Clin Psychol.

[42] Eaton TT, Abeles N, Gutfreund MJ (1988). Therapeutic alliance and outcome: impact of treatment length and pretreatment symptomatology. Psychotherapy.

